# Resolutions Adopted by the New York Institute of Stomatology at a Meeting Held on May 3rd, 1898

**Published:** 1898-07

**Authors:** E. A. Bogue, Geo. A. Wilson


### Resolutions Adopted by the New York Institute of Stomatology at a Meeting Held on May 3rd, 1898.

WHEREAS, It is evident to us that the conditions required for professional progress are now the same as they were when our honored predecessors established a Periodical Journal, a Society and a College, as means of interchanging ideas, disseminating knowledge, abrogating secrecy, and raising the required standard of education; and

WHEREAS, Our periodical literature cannot subserve its highest function if owned by manufacturing and supply houses, notwithstanding their enterprise, because their interests are not always identical with all lines of professional advancement; and

WHEREAS, The common practices of withholding knowledge concerning the formulae of compounds and of patenting inventions and discoveries, operate to prevent the general diffusion of information, and to deny freedom, to put new ideas into tangible form; therefore it is hereby

*Resolved*, That we deprecate the use of secret compounds, and would urge dentists everywhere to dispense with their use as quickly as they can find other compounds of known composition to take their place; and it is also

*Resolved*, That dentists who manufacture or have an interest in secret compounds, or who patent their inventions or discoveries relating to the treatment of deceased or abnormal conditions of the human body, are retarding the progress of our profession; and it is also

*Resolved*, That we would respectfully urge upon all College Faculties and the Association of Dental Examiners of the various States, and the National Dental Association the need of considering present professional conditions, and of adopting such measures as will bring about a change in the views and practices of dentists and students, in regard to journalism, and to secrets and patents. We would hereby entreat all dentists to support by all means in their power the journals we have, not owned by supply houses, to the end that they may be made stronger, and have the means to do their work of spreading knowledge and building up professional moral character more effectually; and it is hereby

*Resolved*, That copies of these resolutions, signed by the President and Secretary, be sent to the Faculty of each Dental College and to the Association of Dental Faculties, and to the Board of Examiners of each State, and to the National Dental Association.

E. A. BOGUE, *President.*
GEO. A. WILSON, *Secretary.*

